# Relationship between metabolic syndrome and moderate-to-vigorous physical activity among adults 18 years old and over

**DOI:** 10.1371/journal.pone.0258097

**Published:** 2021-10-13

**Authors:** Ji-Hoon Cho, Jupil Ko, Seung-Taek Lim

**Affiliations:** 1 Department of Sport and Leisure Studies, Shingyeong University, Hwaseong, Republic of Korea; 2 Division of Health & Kinesiology, Incheon National University, Incheon, Republic of Korea; 3 Olympic Studies Center, Kangwon National University, Chuncheon, Gangwon-do, Republic of Korea; Temple University School of Medicine, UNITED STATES

## Abstract

The prevalence of metabolic syndrome (MetS) risk factors among the Korean population requires effective health surveillance and examination of the effects of preventative behaviors. Thus, the objective of this study is to evaluate the relationships between the clustering of MetS and MVPA in a large sample of 36,987 Koreans ranging from 20 to 80 years of age. This study recruited a total of 36,987 adults (23,813 males and 13,174 females). All participants were assessed for moderate-to-vigorous physical activity (MVPA) using the Korean version short form of the International Physical Activity Questionnaire (IPAQ). The International Diabetes Federation and the Adult Treatment Panel III criteria for blood pressure, hyperglycemia, low high-density lipoprotein cholesterol (HDL-C), and high triglycerides (TG) defined MetS. Waist circumference (WC) was determined by Asian-Pacific region populations. According to the 150-minute MVPA, there were differences in MetS risk factors in young adult males, and only three factors (WC, HDL-C, and TG) were different males in ≥ 70 years old. In females, there was a difference in MetS risk factors in the elderly, and only three factors (WC, blood pressure, and TG) were different females in ≤ 29 years old. The males who did not met the recommended MVPA had a 1.16 to 3.14 -times increase in the MetS risk factors. The females who did not met the recommended MVPA had a 1.18 to 2.57 -times increase in the MetS risk factors. Our study provides evidence that Korean adults who do not engage in recommended MVPA levels increase the odds ratio for each of the MetS risk factors when compared to those who meet the recommendations.

## 1. Introduction

The Metabolic Syndrome (MetS) is commonly defined as the clustering of three or more risk factors including excessive waist circumference, elevated blood pressure, hyperglycemia, low high-density lipoprotein cholesterol (HDL-C), and high triglycerides (TG) [1]. MetS is a set of simultaneous pathological changes that increase the risk of chronic diseases [2] such as cardiovascular disease [3] and diabetes mellitus [4]. Its main cause is not genetic but belongs to modifiable risk factors such as environmental and behavioral factors [5, 6].

Current epidemiological studies show that moderate-to-vigorous physical activity (MVPA) is associated with reducing the risk of MetS development regardless of aerobic fitness and obesity [7–12]. Additionally, the positive improvement of blood pressure suggests a correlation between physical activity and hypertension due to the influence of physical activity/exercise interventions normotensives and prehypertensives [13]. 150 min of physical activity each week was associated with low diastolic blood pressure, high HDL cholesterol, and low total cholesterol [14]. Other studies have also shown that regular physical activity prevents the development of MetS and chronic diseases such as type 2 diabetes [15, 16].

Regular physical activity means the participation in physical activity above a certain level in order to achieve positive effects from physical activity. The American College of Sports Medicine (ACSM) physical activity guidelines recommends participating in moderate intensity physical activity for 150 min per week or 75 min per week for vigorous intensity for health promotion and maintenance in adults [17, 18]. Thus, it is advisable to engage in regular physical activity to reduce or prevent the risk of MetS or comorbidities [8, 19].

For Koreans, 29% of men and 32.9% of women experience MetS, and considering the rapid development of the Korean economy over the past 30 years, the prevalence of metabolic syndrome is expected to continue to increase [20]. Therefore, developing effective and affordable strategies to combat MetS will be of great individual and societal importance. It is important for Korean population health surveillance that the prevalence of MetS risk factors and the effects of preventative behaviors be examined in the Korean context. Thus, the objective of this study is to evaluate the relationships between the clustering of metabolic risk factors and MVPA in a large sample of 36,987 Koreans aged 20–80 years old.

## 2. Materials and methods

### 2.1. Participation

For the present study, 36,987 participants (male = 23,813, female = 13,174) were recruited. All participants were recruited from Nasaret International Hospital (Incheon, South Korea) from January 2018 to December 2019.

The data set was drawn from a retrospective cohort based on Nasaret International Hospital Medical Informatics Data (NIHMID), and separate patient recruitment procedures were not carried out. As the data were de-identified, the informed consent of the subject was not applicable. In the NIHMID, de-identified join keys replacing personal identifiers are used to secure ethical clearance. Therefore, the researcher cannot receive informed consent from individual patients for the use of personal information. However, the use of NIHMID for research purposes requires approval from the institutional review board. This study was approved by the Institutional Review Board at Kangwon National University Institutional Review Board (KWNUIRB-2019-07-009-003).

The characteristics of the participants are shown in Table 1.

**Table 1:** The characteristic of the subjects.

	Height (cm)	Weight (kg)	BMI (kg/m^2^)
Male (n = 23813)			
≤ 29 years (n = 3015)	174.22 ± 6.11	74.58 ± 12.35	24.53 ± 3.59
30–39 years (n = 5782)	174.78 ± 5.97	78.06 ± 12.03	25.52 ± 3.49
40–49 years (n = 7320)	172.99 ± 5.77	75.59 ± 10.75	25.23 ± 3.15
50–59 years (n = 5136)	170.14 ± 5.57	71.57 ± 9.23	24.70 ± 2.76
60–69 years (n = 2202)	167.92 ± 5.44	68.93 ± 8.44	24.42 ± 2.60
≥ 70 years (n = 358)	165.63 ± 5.63	64.52 ± 8.06	23.51 ± 2.66
Female (n = 13174)			
≤ 29 years (n = 826)	162.43 ± 5.39	58.26 ± 10.14	22.08 ± 3.62
30–39 years (n = 1301)	162.67 ± 5.17	60.21 ± 10.58	22.75 ± 3.84
40–49 years (n = 4462)	160.23 ± 5.24	60.25 ± 9.67	23.47 ± 3.64
50–59 years (n = 4275)	157.56 ± 5.01	59.04 ± 8.34	23.78 ± 3.13
60–69 years (n = 1800)	155.60 ± 5.05	58.82 ± 8.07	24.29 ± 3.11
≥ 70 years (n = 510)	152.00 ± 5.27	57.03 ± 7.68	24.68 ± 3.01

BMI, body mass index.

### 2.2. Measurement of anthropometric and blood pressure

The participant’s anthropometric variables measured included body mass, height, and waist circumference (WC). Body mass and height were measured to the nearest 0.1 kg and 0.1 cm, respectively, using a body composition analyzer (Inbody 720, Body Composition Analyzer; Biospace, Seoul, Korea). Body mass index (BMI) was calculated as weight in kilograms divided by the square of height in meters. WC (cm) was measured using a steel measuring tape, with measurements made midway between the lowest rib and the iliac crest in a horizontal plane.

Blood pressure was measured 3 to 4 times after resting quietly in the sitting position for a minimum of 10 minutes. All tests were measured by trained examiner.

### 2.3. Moderate-to-vigorous physical activity (MVPA)

The computerized Korean version short form of IPAQ used in this study was entirely based on the long, self-administered, usual week-long IPAQ found in the IPAQ manual of operation. The 7-item IPAQ identified the total minutes over the last 7 days spent on moderate-to-vigorous physical activity (MVPA), walking physical activity, and inactivity [21]. The questionnaire collects information on time (i.e. number of sessions and average time per session) spent walking, participating in moderate intensity physical activity, participating in vigorous-intensity physical activity and sitting, on weekdays and weekend days. Questions regarding participation in moderate and vigorous physical activity were supplemented by concrete examples of activities commonly performed. Data from the questionnaire were summed within each item (i.e., vigorous-intensity activity, moderate-intensity activity, walking) to estimate the total amount of time spent in physical activity per week. The reliability of the Korean version short form of IPAQ was validated; Spearman rho coefficients and kappa values of the test-retest reliability were 0.43–0.65 and 0.37–0.62, respectively [22].

Based on the self-reported time spent on MVPA, subjects were categorized as being sufficiently or insufficiently active according to the American College of Sports Medicine (ACSM)/Centers for Disease Control and Prevention (CDC) guidelines [23], i.e. accumulating at least 150 min of moderate-intensity activity per week.

### 2.4. Blood collected and analyzed

Fasting venous blood samples were collected from all participants. Fasting was maintained for 8 h. Blood samples were collected the next day after participants had acquired enough sleep and had refrained from as much radical movement as possible. The samples were immediately centrifuged at 3,500 g at 4°C for 10min and analyzed within 24 h. Serum levels of total triglycerides (TG), high-density lipoprotein cholesterol (HDL-C), and glucose were measured by biochemical automatic analyzer using commercial kits (Hitachi 7180, Tokyo, Japan) according to the manufacturer’s protocol.

### 2.5. Components of metabolic syndrome risk factors

MetS risk factors were defined using the National Cholesterol Education Program Adult Treatment Panel III criteria [24]. Criteria specific to Koreans, Asian-Pacific region populations were used to determine WC [25]. Accordingly, each risk factor was categorized and analyzed.

1. Waist circumference: ≥ 90 cm in males and ≥ 85 cm in females
2. Blood pressure: systolic blood pressure ≥ 130 mmHg or diastolic blood pressure ≥ 85 mmHg
3. Fasting glucose: ≥ 100 mg/dL
4. HDL-C: < 40 mg/dL in males and < 50 mg/dL in females
5. Triglycerides: ≥ 150 mg/dL

### 2.6. Statistical analysis

The SPSS statistical package version 25.0 for Windows (SPSS, Inc., Chicago, IL, USA) was used to perform all statistical evaluations. Means and standard deviations were computed for all variables. Binary logistic regression analyses were performed to examine the independent and joint associations of sufficient MVPA level with MetS risk factors. The odds ratios (ORs) and 95% confidence intervals (CIs) were calculated for these associations. In the analysis of the joint association, the group that met the recommended MVPA level (150 min/wk) was regarded as the reference group. MetS risk factors were further analyzed for significant difference among the aged groups using a One-Way ANOVA, and Post-hoc analysis (Bonferroni) was used to compare specific differences when significance was found. All statistical significance was accepted at the 0.05 level.

## 3. Results

### 3.1. The MetS risk factors according to gender and age

The MetS risk factors according to gender and age are shown in Tables 2 and 3. The relative MetS risk factors such as WC, SBP, DBP fasting glucose, HDL-C and TG were significantly different between MVPA levels in the group ≤ 29 years and over, except males ≥ 70 years. A significant difference was found in the male group ≥ 70 years in WC (P <0.001), HDL-C (P <0.001) and TG (P <0.01). The relative MetS risk factors such as WC, SBP, DBP fasting glucose, HDL-C, and TG were significantly different between MVPA levels in females 40 years old and over. In the group of females ≤ 29 years and in the 30–39 years old female group, significantly different was found in WC (P <0.01), SBP (P <0.001), DBP (P <0.001), and TG (P <0.01).

**Table 2:** Metabolic syndrome risk factors according to ages (male).

		WC	SPB	DPB	Fasting glucose	HDL-C	TG
≤ 29 years	≥ 150 MVPA (n = 1512)	81.19 ± 8.10***	119.0 ± 11.4**	73.71 ± 8.18***	92.23 ± 8.60***	58.34 ± 11.84***	93.50 ± 57.46***
	< 150 MVPA (n = 1503)	82.62 ± 9.85	120.3 ± 10.5	75.38 ± 7.91	93.65 ± 10.1	57.37 ± 11.88	104.67 ± 57.74
30–39 years	≥ 150 MVPA (n = 2419)	84.04 ± 7.82***	121.6 ± 10.3***	76.85 ± 8.14***	95.42 ± 13.6***	55.94 ± 12.16***	121.3 ± 71.4***
	< 150 MVPA (n = 3363)	86.24 ± 9.21	122.9 ± 11.3	78.39 ± 8.84	96.83 ± 14.6	53.35 ± 11.54	139.0 ± 73.9
40–49 years	≥ 150 MVPA (n = 3043)	83.77 ± 7.14***	123.9 ± 11.8**	79.39 ± 9.02***	99.78 ± 16.9**	54.82 ± 12.10***	137.3 ± 74.9***
	< 150 MVPA (n = 4277)	85.40 ± 8.27	124.9 ± 12.3	80.62 ± 9.54	101.3 ± 21.4	52.96 ± 11.30	148.6 ± 75.5
50–59 years	≥ 150 MVPA (n = 2253)	83.37 ± 6.64***	125.8 ± 12.7*	79.80 ± 8.86*	103.6 ± 18.2***	55.17 ± 12.28***	126.8 ± 69.9***
	< 150 MVPA (n = 2883)	84.74 ± 7.58	126.7 ± 13.0	80.43 ± 9.14	106.3 ± 25.1	53.00 ± 12.20	143.8 ± 75.6
60–69 years	≥ 150 MVPA (n = 1049)	83.52 ± 6.51***	128.1 ± 12.6**	77.96 ± 8.19***	106.6 ± 20.2***	55.52 ± 12.44***	114.4 ± 61.4***
	< 150 MVPA (n = 1153)	85.42 ± 7.52	129.8 ± 14.0	79.41 ± 9.56	109.3 ± 26.8	52.95 ± 12.52	129.6 ± 69.0
≥ 70 years	≥ 150 MVPA (n = 157)	82.03 ± 6.66***	130.7 ± 11.8	73.80 ± 7.52	103.4 ± 17.5	54.03 ± 13.74***	100.2 ± 51.2**
	< 150 MVPA (n = 201)	85.58 ± 7.88	132.1 ± 14.2	74.25 ± 9.32	105.6 ± 21.4	49.23 ± 11.41	119.0 ± 60.6
Post-hoc	≥ 150 MVPA (n = 10433)	a, b, c, d, e, g, i	a, b, c, d, e, f, g, h, i, j, k, l, m, n	a, b, c, d, e, f, g, h, i, k, l, m, n, o	a, b, c, d, e, f, g, h, i, j, k, m,	a, b, c, d, e, f	a, b, c, d, f, i, j, k, l, m, n
	< 150 MVPA (n = 13380)	a, b, c, d, e, f, g, j, m, n	a, b, c, d, e, f, g, h, i, j, k, l, m, n	a, b, c, d, f, g, h, i, k, l, m, n, o	a, b, c, d, e, f, g, h, i, j, k, m,	a, b, c, d, e, i, l, n, o	a, b, c, d, f, h, i, k, l, m, n

* *P* < 0.05,

** *P* < 0.01,

*** *P* < 0.001; significantly different in the between aged (each MVPA level groups).

P-value was analyzed by independent t-test.

a = significant between ≤ 29 and 30–39, b = significant between ≤ 29 and 40–49, c = significant between ≤ 29 and 50–59, d = significant between ≤ 29 and 60–69, e = significant between ≤ 29 and ≥ 70, f = significant between 30–39 and 40–49, g = significant between 30–39 and 50–59, h = significant between 30–39 and 60–69, i = significant between 30–39 and ≥ 70, j = significant between 40–49 and 50–59, k = significant between 40–49 and 60–69, l = significant between 40–49 and ≥ 70, m = significant between 50–59 and 60–69, n = significant between 50–59 and ≥ 70, o = significant between 60–69 and ≥ 70.

**Table 3:** Metabolic syndrome risk factors according to ages (female).

		WC	SPB	DPB	Fasting glucose	HDL-C	TG
≤ 29 years	≥ 150 MVPA (n = 387)	69.42 ± 7.57**	109.4 ± 11.2***	69.33 ± 7.98***	88.82 ± 6.94	68.95 ± 14.4	66.03 ± 33.0**
	< 150 MVPA (n = 439)	70.95 ± 8.41	114.6 ± 9.07	71.89 ± 7.68	89.91 ± 12.9	68.96 ± 15.4	74.89 ± 42.5
30–39 years	≥ 150 MVPA (n = 499)	72.34 ± 8.90**	113.3 ± 10.7***	72.07 ± 7.83***	88.87 ± 12.8**	66.81 ± 14.0	75.06 ± 41.7**
	< 150 MVPA (n = 802)	73.93 ± 8.98	117.2 ± 9.02	74.48 ± 8.09	91.39 ± 15.2	66.41 ± 14.4	83.84 ± 48.8
40–49 years	≥ 150 MVPA (n = 1726)	73.76 ± 8.15***	117.7 ± 11.9***	74.31 ± 9.06***	93.96 ± 12.8**	66.18 ± 14.4***	84.53 ± 49.2***
	< 150 MVPA (n = 2736)	75.60 ± 8.82	119.6 ± 11.9	75.48 ± 8.93	95.12 ± 15.3	63.63 ± 13.9	96.04 ± 56.7
50–59 years	≥ 150 MVPA (n = 1788)	75.29 ± 7.50***	121.9 ± 13.6***	76.20 ± 9.18***	97.71 ± 14.3***	64.10 ± 14.3***	100.7 ± 57.3***
	< 150 MVPA (n = 2487)	77.02 ± 8.16	123.7 ± 13.5	77.28 ± 8.97	99.47 ± 17.5	62.57 ± 13.6	107.5 ± 60.2
60–69 years	≥ 150 MVPA (n = 788)	77.76 ± 7.31***	126.4 ± 12.9*	76.43 ± 8.32*	101.4 ± 17.3*	61.39 ± 13.5*	105.2 ± 54.2**
	< 150 MVPA (n = 1012)	79.73 ± 8.12	128.0 ± 14.1	76.93 ± 8.87	102.7 ± 19.9	59.82 ± 12.9	114.7 ± 61.2
≥ 70 years	≥ 150 MVPA (n = 173)	80.81 ± 7.23**	128.9 ± 13.2***	73.53 ± 7.52**	102.3 ± 13.7*	58.33 ± 12.4*	107.1 ± 47.7*
	< 150 MVPA (n = 337)	82.68 ± 7.79	135.4 ± 14.8	76.09 ± 8.98	106.1 ± 23.2	55.51 ± 11.4	118.3 ± 59.9
Post-hoc	≥ 150 MVPA (n = 5361)	a, b, c, d, e, f, g, h, i, j, k, l, m, n, o	a, b, c, d, e, f, g, h, i, j, k, l, m, n	a, b, c, d, e, f, g, h, j, k, n, o	b, c, d, e, f, g, h, i, j, k, l, m, n	b, c, d, e, g, h, i, j, k, l, m, n	b, c, d, e, f, g, h, i, j, k, l
	< 150 MVPA (n = 7813)	a, b, c, d, e, f, g, h, i, j, k, l, m, n, o	a, b, c, d, e, f, g, h, i, j, k, l, m, n, o	a, b, c, d, e, g, h, j, k	b, c, d, e, f, g, h, i, j, k, l, m, n, o	a, b, c, d, e, f, g, h, i, k, l, m, n, o	b, c, d, e, f, g, h, i, j, k, l, m, n

* *P* < 0.05,

** *P* < 0.01,

*** *P* < 0.001; significantly different in the between aged (each MVPA level groups).

P-value was analyzed by independent t-test.

a = significant between ≤ 29 and 30–39, b = significant between ≤ 29 and 40–49, c = significant between ≤ 29 and 50–59, d = significant between ≤ 29 and 60–69, e = significant between ≤ 29 and ≥ 70, f = significant between 30–39 and 40–49, g = significant between 30–39 and 50–59, h = significant between 30–39 and 60–69, i = significant between 30–39 and ≥ 70, j = significant between 40–49 and 50–59, k = significant between 40–49 and 60–69, l = significant between 40–49 and ≥ 70, m = significant between 50–59 and 60–69, n = significant between 50–59 and ≥ 70, o = significant between 60–69 and ≥ 70.

### 3.2. Associations of IPAQ physical activity with MetS risk factors

Table 4 shows the independent associations of physical activity amount with MetS risk factors in males and females. The prevalence of MetS risk factors in males not engaging in the recommended level of MVPA increased 1.88–3.42 fold, with the highest in males ≥ 70 years of age (WC: OR = 3.42, 95% CI = 1.90–6.16, HDL-C: OR = 1.98, 95% CI = 1.11–3.51 and TG: OR = 1.88, 95% CI = 1.09–3.25). The prevalence of MetS risk factors in females not engaging in the recommended level of MVPA increased 1.64–2.57 fold, with the highest in females ≤ 29 years of age (blood pressure: OR = 2.57, 95% CI = 1.34–4.92 and fasting glucose: OR = 1.64, 95% CI = 1.00–2.68).

**Table 4:** Independent associations of IPAQ physical activity time with metabolic syndrome.

	Male		Female	
	OR (95% CI)	p-value	OR (95% CI)	p-value
Physical activity				
Engaging in 150 min MVPA per week	1.00		1.00	
Not engaging in 150 min MVPA per week ≤ 29 years,				
Waist circumference	1.37 (1.13–1.66)	.001	1.64 (0.88–3.07)	.122
Blood pressure	1.10 (0.92–1.31)	.286	2.57 (1.34–4.92)	.004
Fasting glucose	1.48 (1.22–1.78)	<.001	1.64 (1.00–2.68)	.050
HDL-cholesterol	1.44 (1.01–2.05)	.046	1.49 (0.89–2.50)	.130
Triglyceride	1.32 (1.08–1.61)	.007	1.63 (0.77–3.45)	.200
30~39 years				
Waist circumference	1.49 (1.32–1.68)	<.001	1.19 (0.83–1.69)	.347
Blood pressure	1.16 (1.03–1.30)	.013	1.67 (1.13–2.46)	.009
Fasting glucose	1.17 (1.04–1.31)	.009	1.33 (0.93–1.92)	.113
HDL-cholesterol	1.39 (1.12–1.71)	.002	1.39 (0.96–2.01)	.083
Triglyceride	1.63 (1.45–1.83)	<.001	2.04 (1.27–3.28)	.003
40~49 years				
Waist circumference	1.53 (1.36–1.71)	<.001	1.50 (1.24–1.81)	<.001
Blood pressure	1.15 (1.04–1.26)	.005	1.18 (1.01–1.37)	.036
Fasting glucose	1.10 (1.00–1.21)	.054	1.08 (0.94–1.25)	.291
HDL-cholesterol	1.16 (0.97–1.38)	.105	1.48 (1.23–1.78)	<.001
Triglyceride	1.27 (1.15–1.39)	<.001	1.66 (1.36–2.02)	<.001
50~59 years				
Waist circumference	1.55 (1.35–1.79)	<.001	1.70 (1.42–2.04)	<.001
Blood pressure	1.07 (0.95–1.19)	.257	1.12 (0.98–1.27)	.098
Fasting glucose	1.07 (0.96–1.19)	.249	1.20 (1.06–1.36)	.005
HDL-cholesterol	1.46 (1.19–1.79)	<.001	1.09 (0.92–1.29)	.338
Triglyceride	1.54 (1.37–1.73)	<.001	1.21 (1.03–1.42)	.022
60~69 years				
Waist circumference	1.78 (1.45–2.19)	<.001	1.61 (1.28–2.04)	<.001
Blood pressure	1.11 (0.94–1.31)	.243	1.00 (0.83–1.20)	.978
Fasting glucose	1.05 (0.89–1.24)	.582	1.12 (0.93–1.35)	.251
HDL-cholesterol	1.53 (1.15–2.05)	.004	1.22 (0.97–1.54)	.088
Triglyceride	1.48 (1.22–1.79)	<.001	1.49 (1.17–1.90)	.001
≥ 70 years				
Waist circumference	3.42 (1.90–6.16)	<.001	1.38 (0.94–2.04)	.102
Blood pressure	1.00 (0.66–1.53)	.996	1.84 (1.27–2.67)	.001
Fasting glucose	1.16 (0.76–1.76)	.488	0.93 (0.64–1.34)	.693
HDL-cholesterol	1.98 (1.11–3.51)	.020	1.02 (0.68–1.54)	.918
Triglyceride	1.88 (1.09–3.25)	.024	1.30 (0.81–2.10)	.279

OR, odds ratio; CI.

### 3.3. Correlation coefficients between MVPA and MetS risk factors

Table 5 shows the correlation coefficients between MVPA and MetS risk factors. Negative correlation was found between WC (*P* <.001), SPB (*P* <.01), DBP (*P* <.001), Glucose (*P* <.001), and TG (*P* <.001) with MVPA. Additionally, positive correlation was found between HDL-C (*P* <.001) and MVPA.

**Table 5:** Pearson’s correlation coefficients (n = 36,987).

	WC	SBP	DBP	Fasting glucose	HDL-C	TG
MVPA	-.050***	-.014**	-.040***	-.033***	.055***	-.071***

MVPA: ~~~; WC: waist circumference; SPB: systolic blood pressure; DBP: diastolic blood pressure; HDL-C: high density lipoprotein -cholesterol; TG: triglyceride.

** *P* <.01,

*** *P* <.001.

## 4. Discussion

The prevalence of metabolic syndrome is increasing every year, regardless of age [26]. The incidence of MetS in Koreans has increased by 0.6% every 10 years since 1998 [27]. MetS is a cluster of cardiovascular risk factors such as abdominal obesity, dyslipidemia, hypertension and hyperglycemia [28]. To prevent MetS and cardiovascular diseases, adults 18 years of age and older should do at least 150 minutes a week of moderate-to-vigorous intensity aerobic physical activity [29]. Systematic evaluation of the independent contributions of MVPA to MetS risk factors for Koreans is lacking. Therefore, the relationship between the risk factors of MetS in Koreans and MVPA for 150 min per week was presented.

In this study, it was demonstrated that for adult males over the age of 18, all MetS risk factors are associated with the amount of MVPA of 150 min a week. The participating Korean male adults who met the recommended MVPA had reduced MetS risk factors compared to those who did not engage in the recommended MVPA. However, there was no difference in blood pressure and fasting glucose in the group ≥ 70 years old. Most previous studies show that the elderly have increased blood pressure in winter compared to summer [30]. Since we did not recruit and measure targets over a specific period of time, it is assumed that this reason did not show any differences in blood pressure in the group of males ≥ 70 years old. However, unlike males, females did not show a difference in fasting glucose and HDL-C in the ≤ 29 years group. Nolan et al. [31] reported that in those with a higher prevalence of MetS, raised triglyceride levels would be observed; however, HDL-C did not change in young adults. Low HDL is not universally exhibited in all young adults with at least one MetS component [31]. Horst et al. [32] suggested a sex-specific association of metabolic syndrome with various inflammatory parameters and showed a strong sex-dependent association of metabolic syndrome. Moreover, Yi and An [33] reported that females experience physiological changes that make them susceptible to metabolic syndrome with aging, with postmenopausal women having more than double the prevalence of metabolic syndrome compared to premenopausal women. In this study, according to the 150-min MVPA, there were differences in MetS risk factors in young adult males, while only three factors (WC, HDL-C, and TG) were different in the ≥ 70 years male group. In the case of females, there was a difference in MetS risk factors in the elderly females, and only three factors (WC, blood pressure, and TG) were different in the ≤ 29 years female group.

The high WC may be the cause agent for all components of the MetS [34]. WC was a better predictor of MetS as compared to other obesity indices such as BMI and WHR in both men and women [35]. The rate of mortality increased across WC in males with two or more MetS risk factors [36]. In this study, the probability of increasing WC of Korean adults who did not engage in recommended MVPA levels was significant increase in male across all ages. In particular, in the ≥ 70 years male group, WC increased by about 3.42 times. For females between the ages of 40 and 69, the WC increase was about 1.5 to 1.7 times. An increase in physical activity was associated with the least amount of gain in weight and change in waist circumference over time, while decrease in physical activity was associated with the highest weight gain and waist circumference gain over time [37]. WC compared to total body fat correlates significantly better with SBP and DBP [38]. In this study, the ≤ 29 years female group had the blood pressure highest prevalence rate of 2.57 times. Moreover, in the ≥ 70 years female group, blood pressure increased by about 1.84 times. For males between the ages of 30 and 49, the blood pressure increase was about 1.15 to 1.16 times. The dependency between MVPA and blood pressure or hypertension risk remained present over all age groups, suggesting that more MVPA is more beneficial [39]. Patients with obesity or metabolic syndrome tend to have low HDL-C because of lower lipoprotein lipase activity and TG enrichment [40]. Physical activity was associated with lower prevalence of the metabolic syndrome, and adults who took more physical activity time had lower waist circumference, higher HDL-C levels and lower TG [41]. In this study, the probability of increasing TG in Korean adults who did not engage in recommended MVPA was significantly increased in male across all ages. HDL-C showed significant differences in all ages except for the 40–49 years group. On the other hand, the probability of increasing HDL-C (1.48 times) and TG (1.66 times) of Korean adults who did not engage in the recommended MVPA level was significantly increased in the 40–49 years female group. Fasting glucose as a MetS risk factor did not increase significantly with age and gender, but the prevalence of fasting glucose increased by about 1.17–1.48 times in males in the ≤ 29 years and 30–39 years groups. Zhu et al. [42] reported no significant association between physical activity and MetS risk factors in females after considering covariates like age, race, income level, and education. Kim et al. [43] also reported an inverse association between physical activity and MetS, especially applied to males.

In this study a significant correlation was shown between MVPA and MetS risk factors (Table 5). Individuals with MetS were more likely to engage in inactive behaviors than those without MetS, and the reciprocal was true for active behaviors [44]. Matthews et al. [45] reported that time spent in sedentary behaviors displaces time spent in lower intensity physical activity behaviors, and can result in an overall reduction in physical activity levels. This study also showed that decreased physical activity negatively affected MetS risk factors. Every additional week, which is equivalent to approximately 20 min of moderate or 10 min of vigorous activity, the odds of MetS were approximately 10% lower [46]. Regular aerobic and resistance exercise showed that there was a reversal of MetS in 19% patients and 42% patients had improvements in at least one component of MetS at 12 months [47]. There was an independent effect of regular physical activity or exercise training on the risk of developing the MetS in individuals without the MetS [8]. Gennuso et al. [48] suggested that adults should try to reduce MetS risk factors by keeping a low level of total time spent in sedentary behaviors.

The present study has some limitations and points to suggestions for further research. First, physical activity amounts were solely investigated using the questionnaire. Second, this study did not consider contributing factors such as diseases, smoking, education levels, and incomes. In future studies, not only should the physical activity level be measured objectively using a triaxial accelerometer, but also factors that affect MetS should be considered and investigated.

## 5. Conclusion

In conclusion, we produced strong evidence showing that Korean adults who did not engage in recommended MVPA levels increased the odds ratio for each of the MetS risk factors when compared to those who met the recommendations. The prevalence of MetS due to activity limitation was increased among adults 18 years old and over. Therefore, in order to prevent MetS, physical activity should be increased and guidelines should be presented according to the cause s of restriction on activity, age, and gender. Also, we strongly recommend regular MVPA be performed for more than 150 min a week.
